# Vaccination

**Published:** 1885-12-20

**Authors:** 


VACCINATION.—In the American Public Health Association, recently in session at Washington, Dr. Kingston, of Montreal, commented on the immense damage that had resulted from the anti-vaccination sentiment among the Canadian French. The unfortunate effect, however, of this folly had wrought a great change in the minds of the people, so that the opposition had almost entirely disappeared.

